# Amniotic Membrane and Its Derivatives: Novel Therapeutic Modalities in Liver Disorders

**DOI:** 10.3390/cells12162114

**Published:** 2023-08-21

**Authors:** Mandana Kazem Arki, Kasra Moeinabadi-Bidgoli, Nikoo Hossein-Khannazer, Roberto Gramignoli, Mustapha Najimi, Massoud Vosough

**Affiliations:** 1 Gastroenterology and Liver Diseases Research Center, Research Institute for Gastroenterology and Liver Diseases, Shahid Beheshti University of Medical Sciences, Tehran 1546815514, Iran; mandana.arki@gmail.com; 2 Basic and Molecular Epidemiology of Gastroenterology Disorders Research Center, Shahid Beheshti University of Medical Sciences, Tehran 1546815514, Iran; kasramoein1996@gmail.com; 3 Division of Pathology, Department of Laboratory Medicine, Karolinska Institute, 17177 Stockholm, Sweden; roberto.gramignoli@ki.se; 4 Laboratory of Pediatric Hepatology and Cell Therapy, Institute of Experimental and Clinical Research (IREC), UCLouvain, B-1200 Brussels, Belgium; 5 Department of Regenerative Medicine, Cell Science Research Center, Royan Institute for Stem Cell Biology and Technology, ACECR, Tehran 1665659911, Iran; 6 Experimental Cancer Medicine, Institution for Laboratory Medicine, Karolinska Institute, 17177 Stockholm, Sweden

**Keywords:** human amniotic membrane, amniotic membrane mesenchymal stromal cell, acute liver failure, chronic liver disease, tissue engineering

## Abstract

The liver is a vital organ responsible for metabolic and digestive functions, protein synthesis, detoxification, and numerous other necessary functions. Various acute, chronic, and neoplastic disorders affect the liver and hamper its biological functions. Most of the untreated liver diseases lead to inflammation and fibrosis which develop into cirrhosis. The human amniotic membrane (hAM), the innermost layer of the fetal placenta, is composed of multiple layers that include growth-factor rich basement membrane, epithelial and mesenchymal stromal cell layers. hAM possesses distinct beneficial anti-fibrotic, anti-inflammatory and pro-regenerative properties via the secretion of multiple potent trophic factors and/or direct differentiation into hepatic cells which place hAM-based therapies as potential therapeutic strategies for the treatment of chronic liver diseases. Decellularized hAM is also an ideal scaffold for liver tissue engineering as this biocompatible niche provides an excellent milieu for cell proliferation and hepatocytic differentiation. Therefore, the current review discusses the therapeutic potential of hAM and its derivatives in providing therapeutic solutions for liver pathologies including acute liver failure, metabolic disorders, liver fibrosis as well as its application in liver tissue engineering.

## 1. Introduction

The liver is a complex organ responsible for the production of a vast majority of proteins and is one of the main sites (along with kidneys) of detoxification and metabolism of numerous biological elements including lipids and glucose. Such vital and critical functions are aligned with the considerable capacity of this organ for self-regeneration [1]. According to recent reports, chronic liver diseases (CLDs) affect nearly 1.5 billion people around the world. CLDs are mainly caused by non-alcoholic fatty liver disease (NAFLD, 59%), hepatitis B virus (HBV, 29%), and hepatitis C virus (HCV, 9%) [2]. Acute liver failure (ALF), which is a rapid and devastating loss of hepatic function, reportedly affects 2000–3000 people per year in the USA [3]. Unfortunately, although being the main treatment for end-stage liver diseases, orthotopic liver transplantation is still associated with major challenges such as donor shortage which limits its wide accessibility, high costs, high invasiveness, life-long immunosuppression, and risk of rejection [4,5]. These challenges encourage scientists to develop novel therapeutic approaches to overcome these impediments. Cell therapy is a novel therapeutic approach based on transplantation of intact, living cells with the ability of differentiation and secretion of beneficial molecules [6,7].

**Figure 1:**
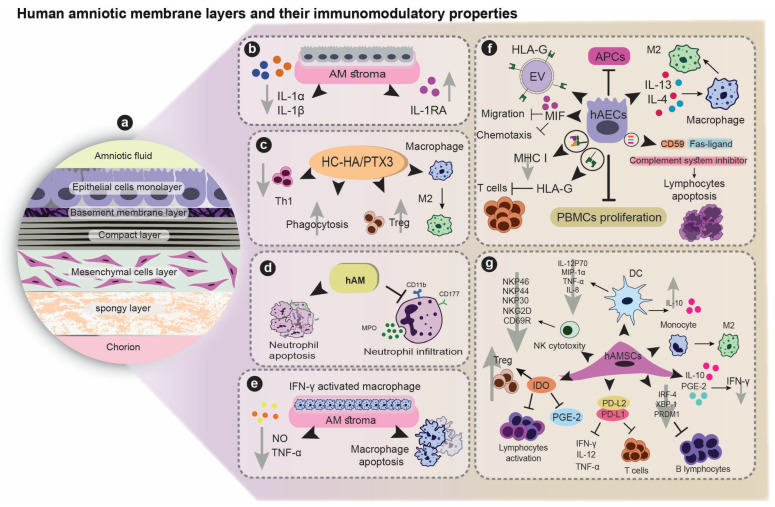
Human amniotic membrane (hAM) and immunomodulatory features of its different layers: (**a**) Graphical representation of hAM which is made up of five layers that are ordered from the inside (amniotic fluid) to the outside (chorion); (**b**) hAM stroma reduces inflammatory cytokines while increasing the expression of the IL-1 receptor antagonist IL-1Ra; (**c**) Heavy chain hyaluronan pentraxin 3 (HC-HA/PTX3) induces macrophage polarization to M2 and promotes phagocytosis of apoptotic cells. HC-HA/PTX3 could suppress Th1 and induce expansion of the Treg cells; (**d**) hAM has the capacity to function as a physical barrier to neutrophil infiltration and to trigger their apoptosis; (**e**) hAM stroma enhances IFN-γ stimulated macrophages’ apoptosis and reduces the production of TNF-α and NO; (**f**) hAECs express a low level of MHC I on their surface. By expressing CD59, Fas-ligand, and complimentary system inhibitor on their surfaces, hAECs can cause lymphocyte apoptosis. hAECs can also inhibit the proliferation of PBMCs and APCs. MIF can be secreted by AECs to impede macrophage migration and chemotaxis. hAECs produce IL-4 and IL-13, which cause M2 phenotype polarization. It also expresses HLA-G on its surface to suppress T cell proliferation and CD8+ inactivation. Extracellular vesicles (EVs) derived from hAEC express HLA-G on their surface; (**g**) To suppress NK cell cytotoxicity, hAMCs can be used to downregulate the NKP30, NKP44, NKG2D, and CD69 receptors. hAMSCs can secrete IL-10 and PGE2 to reduce IFN-γ secretion. hAMSCs suppress B lymphocyte proliferation and maturation by downregulating IRF-4, PRDM1, and XBP-ACs produce IDO, which boosts the number of Tregs while suppressing active lymphocytes and PGE2. hAMSCs can alter monocyte polarization to the M2 phenotype. hAMSCs stimulate DCs to secrete anti-inflammatory cytokines while suppressing inflammatory cytokine secretion by DCs such as TNF-α. hAMSCs express PD-L1 and PD-L2 to limit inflammatory cytokines release and to suppress T cell proliferation and differentiation.

The human amniotic membrane (hAM), the innermost layer of the placenta, is composed of two cell types: human amniotic epithelial cells (hAECs) and human amniotic mesenchymal stromal cells (hAMSCs) which are organized around a thick basement membrane [8,9]. hAM is an ideal candidate for tissue engineering and cell-based therapy thanks to its biological properties including anti-inflammatory and immunomodulatory effects, anti-microbial activity, anti-fibrotic properties, and low immunogenicity. Furthermore, hAM and its derivatives are easily available, inexpensive, and not concerned by ethical considerations and tumorigenicity risks like embryonic stem cells [10]. Finally, hAM could also be an excellent scaffold for tissue engineering owing to its unique structure of basement membrane [11]. In recent years, hAM has been extensively investigated and applied as a wound dressing for skin and corneal lesions [12,13]. As hAM is a rich source of growth factors and stem cells with multipotent differentiation capacity, anti-inflammatory and immunomodulatory properties, anti-fibrotic activities, and low antigenicity, it could potentially be applied in a wide range of liver disorders. In this review, a comprehensive and concise insight into the potential applications of hAM in hepatic defects will be discussed. Figure 1 illustrates the immunomodulatory features of the different layers of hAM.

## 2. Amniotic Membrane Anatomy and Components

hAM is a translucent membrane which does not contain any nervous or vascular structures [14,15]. It is composed of five layers organized from the inner to the outer level/part as such: epithelial cell monolayer composed of amniotic epithelial cells (hAECs), acellular basement membrane layer, compact layer, mesenchymal cell layer composed of amniotic mesenchymal stem cells (hAMSCS), and finally, a spongy layer placed in close proximity to the chorion [16]. Both hAECs and hAMSCs are derived from the pre-gastrulation stages of embryogenesis before delineation of the three primary germ layers (mesoderm, ectoderm, and endoderm) [17]. While the compact layer is a dense structure composed of reticular fibers [18], the spongy layer contains type III collagen fibers, hydrated glycoproteins, and glycoproteins [19].

hAECs are cuboidal to columnar and derived from the embryonic epiblast, forming a monolayer which is in direct contact with the amniotic fluid. hAECs are positive for cell surface CD44, desmin, and CA125 epidermal markers [20]. hAMSCs are spread in an extracellular matrix consisting of collagen and laminin and originate from the extraembryonic mesoderm [17]. hAMSCs express mesenchymal cell surface markers CD44, CD90, and the intermediate filament vimentin [21]. The hAM basement membrane which contains type III, IV, and V collagen and proteoglycans such as actin, α-actinin, and spectrin, supports the integrity of hAM while providing a permeable barrier for macromolecules [22].

The hAM is enriched with various growth factors including epidermal growth factor (EGF), hepatocyte growth factors (HGF), keratinocyte growth factor (KGF), fibroblast growth factor (FGF), transforming growth factor-β (TGF-β), insulin-like growth factor (IGF), IGF binding protein-2,3 (IGFBP-2,3), and platelet-derived growth factor (PDGF), granting its unique regenerative and re-epithelialization properties. Indeed, those growth factors play fundamental roles in biological processes such as wound healing, apoptosis, angiogenesis, anti-fibrotic activity, and tissue regeneration [23,24,25].

The hAM has raised great interest in regenerative medicine, thanks to its inability to evoke allogeneic or xenogeneic immunological responses. Aside from the properties of an amniotic cell, its extracellular matrix (ECM) has shown a great potential to be used as a biomaterial in tissue engineering. Different studies have utilized decellularized amniotic membrane scaffolds and have shown considerable anti-inflammatory properties and functional support necessary for cell attachment, migration, and nutrition supply [26,27]. Scaffolds made up from hAM possess unique biomechanical and biodegradability properties and are being investigated for multiple tissue engineering including blood vessels, urothelium, and cartilage regeneration [28].

Amniotic membrane extract (AME) is a soluble derivative of hAM and contains various bioactive molecules such as fibronectin, HGF, EGF, basic FGF (bFGF), TGF-β, and collagen type I, III, IV, and V. AME has been applied for the regeneration of various tissues, especially cornea. Indeed, AME has been reported to facilitate corneal regeneration by promoting the migration, differentiation, and adhesion of corneal epithelial cells while inhibiting their apoptosis [29].

## 3. Immunomodulatory Properties of Human Amniotic Membrane

The anti-inflammatory effects of hAM are mediated by both mechanical impacts and secreted soluble factors [30]. Solomon et al. showed that culturing lipopolysaccharide (LPS)-induced human limbal epithelial cells on hAM stroma diminished the expression levels of interleukin-1α (IL-1α) and IL-1βl inflammatory cytokines while upregulating IL-1 receptor antagonist (IL-1Ra) [31]. AME also reduced inflammation via inhibiting neutrophil infiltration, by functioning as a physical barrier as well as by inducing their apoptosis [32]. A culture of interferon-γ (IFN-γ) activated macrophages on hAM stroma, is associated with a significantly enhanced apoptosis and diminished secretion of tumor necrosis factor-α (TNF-α) and nitric oxide (NO) by those cells [33].

The main influence on hAM’s immunological properties is the amniotic cells population (ACs) (both hAECs and hAMSCs). hAECs express low levels of major histocompatibility complex-I (MHC-I) molecule on their surface, resulting in a notable immune-privileged property [34]. Moreover, it has been demonstrated that hAECs express CD59, a complement system inhibitor, and Fas-ligand, an apoptosis inducer in infiltrating lymphocytes [35]. hAECs have been reported to hamper the proliferation of peripheral blood mononuclear cells (PBMCs) and to inhibit antigen presentation by antigen presenting cells [36]. hAECs could suppress inflammation via the inhibition of macrophages’ migration and chemotaxis as well as the secretion of a migration inhibitory factor (MIF) [37]. ACs have also been reported to inhibit the cytotoxicity of natural killer (NK) cells by downregulating the expression of NKp30, NKp44, NKp46, NKG2D, and CD69 receptors and decreasing the secretion of IFN-γ by activated NK cells; possibly through release of IL-10 and prostaglandin-2 (PGE-2) [38,39]. hAECs have been shown to stimulate their polarization into M2 phenotype consequently to the release of high amounts of IL-4 and 13 [40]. When co-cultured with AC or incubated with their related conditioned medium (CM), M1 macrophages displayed lower expression of co-stimulatory markers (CD80, CD86, and CD40), and induction of T cell response as well as a significant decrease in the number of IFN-γ-producing CD4^+^ T cells. When purified T cells were co-cultured with either M1 macrophages exposed to the CM or M2 macrophages, an increasing number of activated Treg cells was observed. The great advantage of switching to the anti-inflammatory M2 phenotype was confirmed in various in vivo studies, including liver fibrosis, lung fibrosis, and multiple sclerosis mouse models [41,42,43].

On the other hand, hAMSCs exhibit potent immunomodulatory activities. In the co-culture system, hAMSCs significantly diminished the proliferation of B lymphocytes and their maturation. The latter effect was mediated via a downregulation of the expression of interferon regulatory factor 4 (IRF-4), PR/SET domain 1(PRDM1), and X-box binding protein 1 (XBP-1) genes [44]. hAMSCs secrete significant levels of indoleamine 2,3-dioxygenase (IDO), a potent bioactive factor that increases the Treg cell population and suppresses the activated lymphocytes, as well as prostaglandin E-2 (PGE-2) which inhibits the macrophage activation and NK cells cytolysis [45]. Magatti et al. indicated that hAMSCs and their CM could shift the differentiation of monocytes toward an anti-inflammatory M2 phenotype [46,47]. Furthermore, hAMSCs drastically diminished the function of dendritic cells (DCs) by suppressing their generation and maturation as revealed in-vitro [48]. It was also shown that when co-cultured with ACs (cell to cell or transwell system), DCs displayed a lower potential to trigger CD4^+^ and CD8^+^ T cell proliferation while secreting considerable amounts of anti-inflammatory cytokine IL-10 and lower levels of pro-inflammatory cytokines and chemokines TNF-α, IL-12p70, IL-8, and macrophage inflammatory protein-1α MIP-1α [49]. ACs have been shown to express human leukocyte antigen G (HLA-G), a protein attributed in immunomodulatory properties [50]. Cryopreserved hAECs and hAEC-derived extracellular vesicles (EVs) could express membrane-bound HLA-G on their surface, and hAECs could also release this molecule as a soluble protein [51]. hAECs-HLA-G interaction with T cells leads to an inhibition of their proliferation, switching into Treg phenotype, inactivation of CD8^+^ effector T cells, and apoptosis of activated CD8^+^ T cells [51].

One of the critical components of AME responsible for the anti-inflammatory effects is the heavy chain-hyaluronan pentraxin 3 (HC–HA/PTX3). HC–HA/PTX3 has been reported to induce macrophage polarization into M2 phenotype and to promote phagocytosis of apoptotic cells by macrophages which leads to a diminished local inflammation [52]. In-vitro studies revealed that HC–HA/PTX3 complex could suppress type 1 T helper (Th1) CD4^+^ cells and increase the expansion of Treg cells in order to downregulate the alloreactive immune cells. This concept was confirmed in an in-vivo study after showing that corneal allograft rejection was suppressed by sub-conjunctival injection of HC–HA/PTX [53]. In line with this, administration of hAM-extracted HC–HA/PTX3 reduced the infiltration of inflammatory/immune cells in the lacrimal glands and relived the symptoms in a murine model of chronic ocular graft versus the host disease [54]. Interestingly, ACs promote immune tolerance by expressing the immune checkpoint proteins programmed death-ligand 1 and 2 (PD-L1 and PD-L2) [55,56]. The interaction of PD-L1 and PD-L2 with their receptors could prevent the secretion of IFN-γ, TNF-α, and IL-2 inflammatory cytokines and lead to suppressed T cell differentiation and proliferation [55]. Figure 1 illustrates the immunomodulatory properties of AM.

## 4. Anti-Fibrotic Properties of Amniotic Membrane

Fibroblasts play a significant role in the proliferation phase of wound healing. Collagen production by fibroblasts provides structural stability and reduces wound size. Although collagen and glycosaminoglycan’s assembly in the early phase of wound repair is crucial for a proper healing process, it could lead to an irregular fibril arrangement and fibrosis [57]. Liver fibrosis which is caused by various inducers such viral hepatitis, excessive alcohol consumption, and fat deposition can lead to liver dysfunction and failure, and an enhanced risk of liver malignancies [58,59].

Inflammation is a major step in fibrosis development. Inflammatory response stimulates the migration and proliferation of fibroblasts, ECM production, as well as their differentiation into myofibroblasts. Myofibroblasts play a major role in the healing process as they produce ECM proteins, regulate ECM remodeling, and secrete inflammatory and fibrotic agents. In addition, they can cause maladaptive remodeling and tissue malfunction, leading to tissue fibrosis. In order to avoid fibrotic tissue formation, it is critical to prevent the differentiation of fibroblasts into myofibroblasts [60,61]. TGF-β1 is a key factor that stimulates the activation of hepatic stellate cells (HSCs) and their differentiation into myofibroblasts. It induces the production and accumulation of ECM ingredients such as collagen and fibronectin by hepatocytes, hampers matrix metalloproteinases’ (MMPs) digestive function against ECM, and upregulates the generation of inflammatory/fibrogenic factors including PDGF, TNF-α, and IL-1β [62,63].

**Figure 2:**
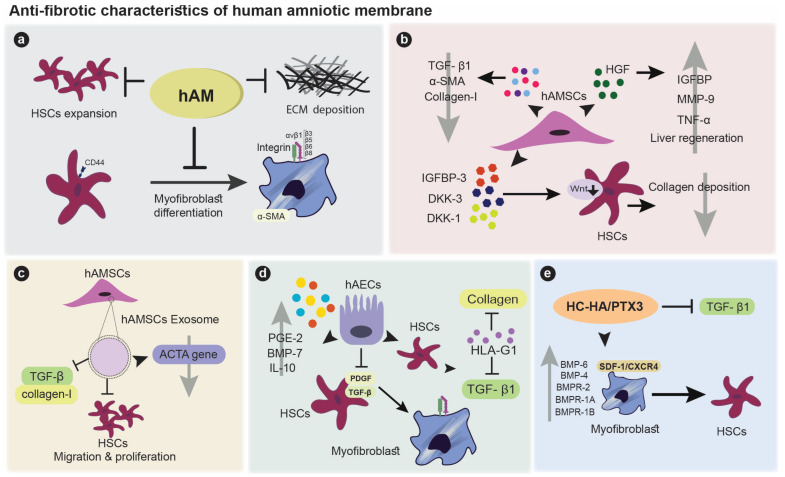
The anti-fibrotic properties human amniotic membrane: (**a**) hAM decreases ECM deposition, HSC expansion, and myofibroblast differentiation; (**b**) hAMCs reduce collagen I, α-SMA, and TGF-β expression while also secreting HGF to promote liver regeneration, IGFBp to limit hepatocyte apoptosis, MMP9 to degrade ECM, and TNF- to inhibit HSC activity. They can secrete IGFBP-3, DKK-3, and DKK-1 to impair Wnt cell signaling and inhibit HSC function with collagen deposition; (**c**) AM-derived exosomes can diminish liver fibrosis by inhibiting HSC proliferation and migration, as well as TGFβ-1 and collagen formation. These exosomes can also suppress the expression of the ACTA gene; (**d**) By inhibiting TGF-β1 and PDGF signaling pathways, hAECs prevent HSC differentiation into myofibroblasts. PGE2, BMP7, and IL-10 are anti-fibrotic substances secreted by hAECs. These cells induce HSCs to produce HLA-G1, which inhibits collagen and TGF-β1 production; (**e**) HC-HA/PTX3 inhibits TGF-β1 secretion and reverses the myofibroblast phenotype by increasing the expression of BMP-4, BMP-6, BMPR1A, BMPR1B, and BMPR2.

The hAM and its derivatives could hamper tissue fibrosis through anti-inflammatory and anti-fibrotic properties. The hAM diminishes ECM deposition and the expansion of HSCs and their differentiation into myofibroblasts as reported in a rat model of liver fibrosis [64]. Activation of HSCs is characterized by an induced expression of smooth muscle *alpha*-actin (α-SMA) and contractile activity. Transplantation of hAMSCs in a rat alleviates fibrosis through the suppression of collagen-I, α-SMA, and TGF-β1 expression. hAMSCs also secrete high levels of HGF, which enhances liver regeneration; insulin-like growth factor binding protein (IGFBP), which inhibits hepatocyte apoptosis; MMP-9, that degrades ECM; and TNF-α, which suppresses HSCs activation [65]. hAECs are able to diminish liver fibrosis via a blockade of TGF-β and PDGF signaling, and an induced secretion of anti-fibrotic factors PGE-2, bone morphogenetic protein-7 (BMP-7), and IL-10 [66]. Their ability to reduce collagen and TGF-β1 synthesis by HSCs via HLA-G1 secretion has also been documented [67]. The same effects were observed when hAM-derived exosomes were investigated [68]. Indeed, amniotic cells, mainly hAMSCs, are able to produce exosomes displaying anti-fibrotic properties. Incubation of HSCs with hAM-derived exosomes results in a significant prohibition of TGF-β1-activated HSCs, inhibition of HSC migration, and profound down-regulation in the expression of fibrosis-associated genes such as α smooth muscle actin (ACTA) [68]. AME is able to reverse the myofibroblasts phenotype into fibroblasts and to inhibit fibrosis [69], an effect mostly attributed to HC-HA/PTX3-mediated TGF-β1 suppression [57]. Such a role of HC-HA/PTX3 is mediated by an upregulation of the expression of BMP-4, BMP-6, BMP receptor1A (BMPR1A), BMPR1B, and BMPR2 consequently to the activation of stromal cell derived factor-1/C-X-C chemokine receptor type 4 (SDF-1/CXCR4) signaling [70]. An equivalent anti-fibrotic effect was shown for hAMSCs in mouse models of liver fibrosis. This was mediated via their secretion of IGFBP-3, Dickkopf-3 (DKK-3), and Dickkopf-1 (DKK-1) which subsequently hamper the Wnt pathway in HSC [71]. Figure 2 illustrates the anti-fibrotic properties of the hAM.

## 5. Amniotic Membrane and Its Application in Liver Diseases

**Figure 3:**
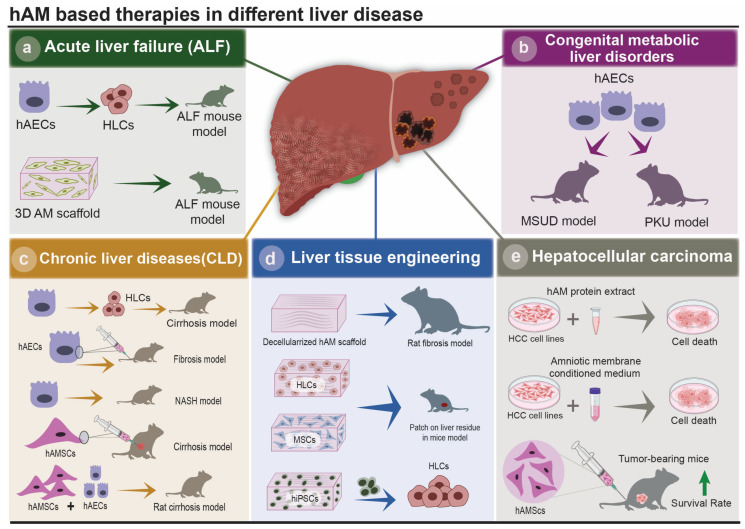
Highlights of the application of human amniotic membrane in liver diseases and liver tissue engineering: (**a**) Use of hAM in acute liver failure (ALF). hAESCs can be transplanted into an ALF animal model; (**b**) Recent research on the use of hAM in congenital metabolic liver diseases: Transplantation of hAECs into mouse models of MSUD and PKU; (**c**) Investigations on the use of hAM in chronic liver disorders (CLD). hAECs able to develop into functional hepatocyte-like cells, were studied in a cirrhosis mouse model. hAECS were injected via the tail vein into immunocompetent animals with fibrosis. and transplanted in a mouse model of NASH. hAMSCs were infused in cirrhotic mice while hAECs and hAMSCs were transplanted in a cirrhotic rat model; (**d**) Current research on the application of AM in liver tissue engineering. Decellularized hAM scaffold was transplanted into a fibrosis rat model. Acellular hAM was employed as a 3D scaffold in combination with HLCs grafted into a mouse model. In mice, MSCs were seeded on acellular hAM utilized as a patch on liver residue. hiPSCs differentiate into functional hepatocyte-like cells when seeded on decellularized AM; (**e**) Recent studies about hAM application in HCC. Both hAM protein extract and conditioned medium were applied on different HCC cell lines in vitro while hAMC suspensions were peripherally infused into tumor bearing mice.

Liver diseases are divided into three general categories: acute, chronic, and metabolic damages. Acute liver diseases are caused by the storage of toxic compounds and result in hepatocyte dysfunction and even hepatic failure. Chronic diseases cause liver inflammation, fibrosis, cirrhosis, and an increased chance of malignancies such as hepatocellular carcinoma. Metabolic disorders are caused by an inherited single structural protein or enzymatic defects [72,73]. The hAM could be utilized as a therapeutic modality for various liver disorders including acute liver failure (ALF), chronic liver diseases (CLD), and congenital disorders thanks to its immunomodulatory performance, anti-fibrotic activity, hepatocyte differentiation capacity, as well as to its ability to be used as a scaffold. Figure 3 summarized the different potential applications of the different layers of the hAM in liver diseases.

## 6. Acute Liver Failure

ALF is a fatal syndrome accompanied by liver dysfunction, hepatocyte necrosis and multiple organ failure [74]. ALF is associated with the dysfunction of humoral and innate immune responses which lead to an acute liver inflammation [75]. ALF is an unpredictable state with more than 2500 cases reported each year in the United States and the condition demands an urgent follow-up for liver transplantation [76]. ALF is caused by a variety of factors, however, the most common causes are viral infections and drug-induced liver toxicity [75]. Although liver transplantation is the main treatment for ALF, its accessibility is severely limited due to lack of sufficient donors and the risk of rejection [77]. To solve this problem, new strategies are currently considered to acquire sufficient functional hepatocytes [78]. Recently, hAECs have been recognized as one of the most promising stem-cell sources for regenerative medicine. These cells are able to differentiate into functional hepatocyte-like cells (HLCs) and then transplanted, a strategy which showed desirable outcomes in a mouse model of ALF. It was demonstrated that transplanted differentiated hAECs exhibit similar metabolic hepatocytic functions such as urea secretion, xenobiotic detoxification, and production of albumin [74]. One of the main hurdles limiting the clinical development of cell therapy for ALF is the low rate of cell retention/engraftment in the liver. To overcome this impediment, hAM has been tested as a 3-dimentional scaffold for cell seeding. In an experiment, hepatic grafts were engineered by seeding adipose-derived stem cells on acellular hAM as a 3-dimensional scaffold. Fabricated hepatic grafts have been shown to express specific human hepatocyte markers including albumin, hepatocyte nuclear factor-4α (HNF-4α), and cytochrome P450 2B. Transplantation of these hepatic grafts in a mouse model of ALF resulted in a significant reduction in the plasma levels of ALF-associated biomarkers and attenuated liver histopathological damage at 8 weeks post-transplantation [79].

## 7. Chronic Liver Diseases

Development of a chronic hepatic injury, characterized by sustained liver inflammation and ECM proteins aggregation, leads to liver fibrosis and results in hepatocyte dysfunction and CLD [80]. HSCs and macrophages play a crucial role in the establishment and perpetuation of liver fibrosis. HSCs secrete ECM proteins and fibrogenic factors. At the same time, macrophages, including resident Kupffer cells and infiltrating monocytes, perform a variety of functions during CLD [81]. The activities of Kupffer cells consist of secreting various pro-inflammatory and pro-fibrotic factors, attracting populations of immune cells to the liver, and phagocyting cell debris [66]. CLD could lead to cirrhosis, portal hypertension, multi-organ failure, hepatic malignancies, and eventually death [2]. Current therapeutic strategies for CLD such as supportive care and pharmacologic treatments have shown inadequate outcomes or are difficult to access. Hence, novel therapeutic modalities such as hAM are currently under consideration [82]. In a study conducted by Manuelpillai et al., hAECs were infused via the tail vein into immunocompetent C57/Bl6 mice with carbon tetrachloride-induced fibrosis. The results showed that single infusion of 2 × 10^6^ hAECs decreased fibrosis and collagen deposition in comparison to the control groups [83]. In a following study with carbon tetrachloride-induced fibrosis mouse models by the same research team, results revealed that after 4 weeks of hAECs transplantation, there was a decrease in hepatic T cell infiltration and macrophage population, as well as a profound diminution in collagen-producing HSCs, which led to the attenuation of fibrosis and hepatic damage [84]. Lin et al. showed that hAECs were able to differentiate into functional hepatocyte-like cells with the capacity of albumin production, induction of P450 enzyme system, bile canaliculi formation, and uptake of low-density lipoprotein from plasma, which illustrates the potential of hAECs to be used as a treatment option for CLD [85]. Transplantation of hAMSCs and hAECs into rat models of cirrhosis resulted in the improvement of hepatic microcirculation, fibrosis attenuation, inflammation and decreased oxidative stress, and consequently, promoted liver function [85].

NAFLD is the most prevalent CLD worldwide that can develop into cirrhosis and hepatocellular carcinoma. Non-alcoholic steatohepatitis (NASH) is the inflammatory form of NAFLD. It is associated with liver fibrosis, oxidative stress, and hepatocyte apoptosis. Transplantation of hAECs in mouse models of NASH resulted in a significant inhibition of HSC proliferation, profound downregulation of TGF-β1 synthesis, and increased production of interferon-β (IFN-β), an anti-fibrotic factor. Furthermore, hAEC treatment suppressed the production of IL-6 and IFN-γ pro-inflammatory cytokines and decreased oxidative stress via an inhibition of neutrophil infiltration and increase in heme oxygenase-1 synthesis [86].

Taken together, hAM and specifically hAECs could be potentially beneficial in the clinical scenarios of CLD, mainly based on the preclinically demonstrated hepatocytic plasticity, anti-oxidative and anti-fibrotic properties.

## 8. Congenital Metabolic Liver Disorders

Congenital metabolic diseases are hereditary disorders caused by a defect of a specific enzyme activity. A possible treatment for these disorders could be diet restriction therapy. However, this is not a lifelong treatment due to the low compliance of patients. Currently, the only curative therapy for severe congenital liver diseases is liver transplantation [87]. As liver transplantation is not available for many patients and is associated with high economic burden and risk of rejection, stem cell therapy is potentially an attractive alternative for the treatment of liver congenital metabolic disorders [88]. Among the available useful cells, hAECs could be an ideal choice due to their unique properties including non-tumorigenicity, differentiation capacity, and non-immune reactivity. Differentiation capacity of hAECs paves the way for hepatocyte replacement therapy and lack of immunogenicity and tumorgenicity boosts their safety profile [89]. Two of the most common congenital liver disorders are maple syrup urine disease (MSUD) and phenylketonuria (PKU). Mutations in the genes which encode enzymes responsible for the metabolism of branched-chain amino acids and branched-chain α-keto acid dehydrogenase (BCKDH) cause MSUD. Mutations in the phenylalanine hydroxylase gene which is involved in phenylalanine metabolism leads to alterations in phenylalanine levels and subsequent symptoms in PKU [90]. hAECs are able to differentiate into functional hepatocytes with normal enzymatic activity and have shown promising outcomes in vivo [91]. It has been reported that hAEC transplantation, as a substitute for hepatocyte-replacement therapy, could significantly improve the metabolism of branched-chain amino acid (BCAA) in sera and the brain in a transgenic murine model of intermediate MSUD [92]. Another study demonstrated that PKU mice transplanted with hAECs had decreased phenylalanine levels in the blood and brain tissues after 100 days follow-up [90]. Taken together, cell therapy with hAECs could be an ideal choice for the treatment of liver congenital disorders due to their availability, capacity to differentiate into functional hepatocytes, lack of ethical concerns, and low risk of rejection and tumorgenicity.

## 9. Amniotic Membrane-Based Scaffolds in Liver Tissue Engineering

With the advent of tissue engineering and regenerative medicine, hAM has been used in a broad range of medical fields. It is an inexpensive, available, and biocompatible material with no ethical issues. It appropriately adheres to the wound surface and reduces scars and pain with a low risk of immunogenicity [93,94]. A significant property of hAM application in tissue engineering is its capacity to provide a proper niche for cell attachment, proliferation, and differentiation.

Decellularized hAM has been applied in blood vessels for tissue engineering [93], cartilage regeneration [95], and urothelium tissue engineering [18]. It has been used as a scaffold for liver regeneration as well. hAM showed an anti-fibrotic effect in the liver of the bile duct ligated rat mainly mediated through paracrine activity [64]. In another study, acellular hAM was utilized as a three-dimensional scaffold and combined with hepatocyte-like cells derived from human adipose stem cells. The mentioned graft was integrated in the liver concomitantly to limit the complications related to an acute liver injury in mice and promoted liver regeneration post-hepatectomy [96]. Finally, bone marrow-derived mesenchymal stromal cells (BM-MSC) were seeded on acellular hAM and used as a patch on 80% hepatectomized mouse livers. The hAM patch seeded with 5 × 10^5^ BM-MSC, has been shown to significantly stimulate liver regeneration and profoundly enhanced the survival of transplanted mice when compared to hAM alone and to BM-MSCs alone [96]. Decellularized AM scaffold could also be an attractive choice for the induction of hepatocytic differentiation. Indeed, seeding human induced pluripotent stem cells (hiPSCs) on decellularized hAM enhances their differentiation into functional hepatocyte-like cells as decellularized hAM ECM composition closely resembles liver in vivo environment and its 3-dimentional structure provides a desirable space for hiPSC adhesion, differentiation, and maturation. Enhanced hiPSCs differentiation into functional hepatocyte-like cells could be demonstrated by an increased synthesis of urea and albumin and augmented activity of CYP3A [97]. hAM scaffold has also been exploited as an ideal substrate for hepatocyte expansion, thanks to its ability to sustain hepatocyte epithelial morphology and functionality (albumin production) for up to two months post-seeding [98].

## 10. Conclusions and Future Perspectives

The hAM is an inexpensive, accessible, and efficient modality for the treatment of various liver diseases. It is non-immunogenic and secretes numerous potent regenerative, anti-fibrotic, and immunomodulatory factors which facilitate liver recovery and regeneration. Multiple preclinical studies have demonstrated the safety and efficacy of hAM in treating liver disorders (Table 1) and should help in the design of appropriate clinical development studies. Paracrine capacity and the post-transplantation survival rate of amniotic cells could be improved by cell preconditioning and genetic modification strategies, which could further ameliorate their therapeutic potential [99]. Amniotic cell-derived exosomes are another appropriate cell-free therapeutic option to consider for the delivery of therapeutic drugs, growth factors, nucleic acids, and regulatory proteins [100,101].

Amniotic cells could also be used as vectors for gene delivery, an emerging approach for the treatment of congenital liver diseases. Gene therapy has been experimentally applied for the treatment of some infectious and inborn disorders of liver diseases and has shown promising outcomes [102]. The biocompatibility, bioavailability and structure of the hAM make it an ideal candidate to be used as a scaffold for tissue engineering. It provides an appropriate environment for cell attachment, proliferation, and differentiation. Decellularized hAM is a thin, elastic, and strong biomaterial that is tolerated well in the body and supports the attachment and proliferation of various cell types. However, batch-to-batch variations are a major hindrance for its wider use, especially in the decellularization process which needs to be addressed. Taken together, biomedical application of hAM and its derivatives is a developing market and could be used for biomedical research and treatment of liver diseases.

**Table 1:** Preclinical studies that evaluate the usefulness of hAM for the treatment of liver disorders.

AM Type	Preclinical Model	Main Results	Reference
**Human amnion epithelial cells (hAEC)**	Immunocompetent CCL_4_ treated mice	Reduced hepatocyte apoptosis/Decreased hepatic inflammation and fibrosis/Cell engraftment	[83]
**Human amniotic membrane** **(hAM)**	Bile duct ligated Rat	Prevented fibrosis progression/Restricted fibrotic degeneration	[103]
**Human amniotic epithelial cells (hAECs)**	thioacetamide-induced chronic mice liver failure	In vitro and in vivo differentiation of hAECs into functional hepatocyte-like cells	[85]
**human amniotic epithelial cells (hAEC)**	Immunocompetent CCL_4_ treated mice	Induced M2 macrophage phenotype/Reduced hepatic fibrosis	[84]
**human amnion epithelial cells (hAECs)**	murine NASH model	Reduced hepatic inflammation and fibrosis by hAECs and its condition medium	[104]
**Human Amnion-Derived Mesenchymal Stem Cells (hAMSCs)**	CCL_4_ treated Rat	Reduced liver fibrosis possibly by inhibition of Kupffer cell and hepatic stellate cell activation	[105]
**Human amniotic epithelial cells (hAECs)**	murine NASH model	increased total levels of anti-fibrotic IFNβ in mice treated with a single dose of hAECs	[86]
**Human Amniotic membrane** **(hAM)**	Bile duct ligated Rat	Reduced liver fibrosis in a surgical animal model of cholestasis	[106]
**Human Amniotic membrane patch**	Bile duct ligated Rat	application of the amniotic membrane around only one hepatic lobe was being more effective in reducing the liver fibrosis	[107]
**Human Amniotic membrane patch**	Wistar albino rats	Applied amniotic membrane to residual liver adversely affected liver regeneration	[108]
**Human Amniotic membrane patch**	Bile duct ligated Rats	AM patch reduce fibrosis by downregulating the profibrotic factor such as TGF-b1 and IL-6	[109]
**Human amniotic mesenchymal stromal cells (hAMSCs)**	Acetaminophen (APAP) induced liver injury mice	hAMSCs suppressed M1 polarization and promoted M2 polarization of Kupffer cells through regulating autophagy	[110]
**amniotic epithelial cells** **(AECs)**	Rat and human Amniotic epithelial cells (AECs)	AECs can respond to proangiogenic signals in vitro and differentiate into HSECs in vivo	[111]
**Human amniotic membrane-derived mesenchymal stem cells (hAMCs)**	Immunocompetent CCL4 treated mice	suppressed activation of hepatic stellate cells, decreased hepatocyte apoptosis and promoted liver regeneration	[112]
**Mice amniotic-fluid-derived stem cells** **(mAFSCs)**	CCL_4_ treated mice	Reduced fibrosis by mAFSCs injection	[113]
**human amniotic membrane-derived mesenchymal stromal (hAMSCs) and epithelial stem cells (hAECs)**	CCL_4_-cirrhotic rats	Rats that received amnion derived stem cells had markedly reduced hepatic inflammation and oxidative stress/improved liver function in rats receiving hAMSCs	[114]
**Human amniotic membrane patch**	Bile duct ligated Rats	AM patch could attenuate the severity of biliary fibrosis for longer periods	[115]
**Human amniotic epithelial cells (hAECs)**	Bile duct ligated Rats	Reduced fibroblast numbers/reduced activation of portal fibroblast	[116]
**Acellular human amniotic membrane**	CCL_4_ treated mice	Functional acellular human amniotic membrane-hepatocytes grafts integrated with the liver decreases the acute liver injury of mice	[79]
**Human amniotic membrane patch**	Bile duct ligated Rats	Cryopreservation maintains the antifibrotic properties of hAM when used as a patch to reduce the liver fibrosis	[64]
**Human amniotic epithelial cell-derived extracellular vesicles (hAEC-EVs)**	CCL_4_ treated mice	hAEC-derived EVs significantly reduced liver fibrosis and macrophage infiltration	[117]
**Human amniotic epithelial stem cells** **(hAESCs)**	CCl_4_-induced ALF mouse	hAESCs were differentiated to hepatocyte-like cells (HLCs) and HLC transplantation via the tail vein could improve the liver function, and significantly prolong the survival of ALF mouse.	[74]
